# Optimized, automated and cGMP-compliant synthesis of the HER2 targeting [^68^Ga]Ga-ABY-025 tracer

**DOI:** 10.1186/s41181-023-00226-y

**Published:** 2023-11-22

**Authors:** Emma Jussing, Mélodie Ferrat, Mohammad M. Moein, Henrik Alfredéen, Tetyana Tegnebratt, Klas Bratteby, Erik Samén, Joachim Feldwisch, Renske Altena, Rimma Axelsson, Thuy A. Tran

**Affiliations:** 1 Department of Radiopharmacy, Karolinska University Hospital, 171 76 Stockholm, Sweden; 2 Department of Oncology and Pathology, Karolinska Institutet, 171 77 Stockholm, Sweden; 3 Karolinska Comprehensive Cancer Center, Karolinska University Hospital, 171 77 Stockholm, Sweden; 4 Affibody AB, 171 65 Solna, Sweden; 5 Department of Medical Radiation Physics and Nuclear Medicine, Karolinska University Hospital, 171 76 Stockholm, Sweden; 6 Department of Molecular Medicine and Surgery, Karolinska Institutet, 171 77 Stockholm, Sweden

**Keywords:** Human Epidermal growth factor Receptor 2, HER2-PET imaging, Gallium-68, ABY-025, Affibody, Breast cancer, HER2 low, [^68^Ga]Ga-ABY-025

## Abstract

**Background:**

The Affibody molecule, ABY-025, has demonstrated utility to detect human epidermal growth factor receptor 2 (HER2) in vivo, either radiolabelled with indium-111 (^111^In) or gallium-68 (^68^Ga). Using the latter, ^68^Ga, is preferred due to its use in positron emission tomography with superior resolution and quantifying capabilities in the clinical setting compared to ^111^In. For an ongoing phase II study (NCT05619016) evaluating ABY-025 for detecting HER2-low lesions and selection of patients for HER2-targeted treatment, the aim was to optimize an automated and cGMP-compliant radiosynthesis of [^68^Ga]Ga-ABY-025.

[^68^Ga]Ga-ABY-025 was produced on a synthesis module, Modular-Lab PharmTracer (Eckert & Ziegler), commonly used for ^68^Ga-labelings. The radiotracer has previously been radiolabeled on this module, but to streamline the production, the method was optimized. Steps requiring manual interactions to the radiolabeling procedure were minimized including a convenient and automated pre-concentration of the ^68^Ga-eluate and a simplified automated final formulation procedure. Every part of the radiopharmaceutical production was carefully developed to gain robustness and to avoid any operator bound variations to the manufacturing. The optimized production method was successfully applied for ^68^Ga-labeling of another radiotracer, verifying its versatility as a universal and robust method for radiosynthesis of Affibody-based peptides.

**Results:**

A simplified and optimized automated cGMP-compliant radiosynthesis method of [^68^Ga]Ga-ABY-025 was developed. With a decay corrected radiochemical yield of 44 ± 2%, a radiochemical purity (RCP) of 98 ± 1%, and with an RCP stability of 98 ± 1% at 2 h after production, the method was found highly reproducible. The production method also showed comparable results when implemented for radiolabeling another similar peptide.

**Conclusion:**

The improvements made for the radiosynthesis of [^68^Ga]Ga-ABY-025, including introducing a pre-concentration of the ^68^Ga-eluate, aimed to utilize the full potential of the ^68^Ge/^68^Ga generator radioactivity output, thereby reducing radioactivity wastage. Furthermore, reducing the number of manually performed preparative steps prior to the radiosynthesis, not only minimized the risk of potential human/operator errors but also enhanced the process’ robustness. The successful application of this optimized radiosynthesis method to another similar peptide underscores its versatility, suggesting that our method can be adopted for ^68^Ga-labeling radiotracers based on Affibody molecules in general.

*Trial registration*: NCT, NCT05619016, Registered 7 November 2022, https://clinicaltrials.gov/study/NCT05619016?term=HER2&cond=ABY025&rank=1

**Supplementary Information:**

The online version contains supplementary material available at 10.1186/s41181-023-00226-y.

## Background

Molecular imaging using positron emission tomography (PET) is a non-invasive imaging technique suitable for the diagnostics of cancer. Radiopharmaceuticals targeting specific receptors make it possible not only to distinguish between malignancy and non-malignancy, but also between malignant cell subtypes expressing possible targets for therapy. One such receptor is the human epidermal growth factor receptor type 2 (HER2) (Tolmachev et al. 2021). Breast cancer is the most common cancer type among women worldwide (Sung et al. 2021). Up to one in eight women will be diagnosed with breast cancer in their lifetime (Harbeck and Gnant 2017). HER2 overexpression is found in 10–15% of breast cancer patients, for whom targeted HER2 therapies have become standard treatment and have increased the progression-free survival by up to 50% (Harbeck and Gnant 2017; Altena et al. 2023; Seban et al. 2023). An increased HER2 expression is found in a variety of malignancies, such as solid tumors of gastroesophageal-, lung-, prostate-, bladder-, and colorectal cancers (Vranić et al. 2021). For optimal patient stratification, invasive biopsies and extensive histopathology are normally required. However, breast cancer is known to have a heterogenous receptor expression, both between tumor lesions and within lesions. This results in a serious limitation using this type of diagnostic methods for determining the correct HER2 status since biopsy samples are only taken from accessible tumor lesions, can hit HER2 negative parts of the lesion and thus not reveal the complete picture of the HER2 expression throughout the whole-body metastatic burden (Sorensen et al. 2014).

To assess HER2 expression status in patients using PET, several radiotracers have been introduced in clinical trials, including long-lived radionuclide labelled antibodies, e.g. zirconium-89/copper-64 trastuzumab or pertuzumab (Wright and Lapi 2013). For same day imaging after administration, an alternative, smaller targeting agent is preferred with faster pharmacokinetics. The Affibody molecule, ABY-025, has demonstrated utility to detect human epidermal growth factor receptor 2 (HER2) in vivo, either labeled with the radionuclide indium-111 (^111^In), fluoride-18 (^18^F), or gallium-68 (^68^Ga) (Sorensen et al. 2014; Sandberg et al. 2017; Glaser et al. 2013). Using one of the two latter, ^18^F or ^68^Ga, is preferred in a clinical setting due to their use in PET with better resolution and quantifying properties, compared to single-photon emission computed tomography (SPECT). These semi-short-lived radionuclides can also be considered a better match to the fast biodistribution and blood kinetics of ABY-025 (Sandstrom et al. 2016). An on-site cyclotron is not needed for access of ^68^Ga, which is an advantage compared to ^18^F. Though, the relatively high cost of the GMP-classified ^68^Ge/^68^Ga-generator must be considered closely, a cost which might not be possible to carry by, or justified for, a single study project. Alongside already ongoing clinical productions and/or parallel study projects, the cost of the ^68^Ga-eluate for the isolated study can be significantly lowered and thus bearable. For ^68^Ga-labelled ABY-025, [^68^Ga]Ga-ABY-025, a manual radiolabeling method has been described for the initial phase 0 studies (Velikyan et al. 2016). This manual method was later transferred to a synthesis module operated method (Velikyan et al. 2019) using fractionation to obtain ^68^Ga from the generator and an addition of peptide in the final formulation step. In current study we describe a further development of the automated cGMP-compliant radiosynthesis method of [^68^Ga]Ga-ABY-025 for an ongoing phase II study (NCT05619016) to evaluate the HER2-status to improve selection of patients for treatment.

[^68^Ga]Ga-ABY-025 was produced on a synthesis module commonly used for ^68^Ga-labelings (Modular-Lab PharmTracer, Eckert & Ziegler). Every part of the radiopharmaceutical production was carefully developed to gain robustness and steps requiring manual interactions were minimized to avoid any operator bound variations to the manufacturing process. The aim was to adjust the previously successfully adopted ^68^Ga-labeling procedure at the Karolinska Radiopharmacy site (Jussing et al. 2021), on this particular synthesizer, to make it suitable for radiolabeling of radiotracers based on Affibody molecules, e.g. [^68^Ga]Ga-ABY-025. We also provide details on quality control (QC) and steps-by-step how the analytical methods are validated.

## Methods

### General

The production of [^68^Ga]Ga-ABY-025 was performed in a class C cleanroom laboratory dedicated for radiopharmaceutical manufacturing. The synthesis module was placed in a hotcell (BBC type, Comecer). A transfer line from the hotcell to a lead shielded product vial hatch made retrieving the product easy and safe in terms of radiation protection for the operator. Both hotcell and product hatch hold the same cleanroom classification as the surrounding laboratory. The product vial was prepared, assembled with the sterile product filter and ventilation filter, in a class A laminar air flow microbiological safety cabinet (Ninolab), moved to the lead shielded product vial hatch and then the transfer line was connected to the inlet of the sterile product filter.

The entire manufacturing process can be divided into different sub-parts: production, pre-release QC, release, post-release QC and certification. Details of the three major software systems and a description of their process role are given in Table 1.

**Table 1:** The three major software systems used for the production of radiopharmaceuticals (e.g. [^68^Ga]Ga-ABY-025) at the Karolinska Radiopharmacy, Karolinska University Hospital

Software	Version	Function/process role
PETra (LabLogic)	2.2.5.49	Laboratory Information Management System (LIMS) used to record and archive all batch data
FMS (Brookhaven)	5.2.1	Facility Monitoring System (FMS) used to record temperature and pressure differentials throughout the cleanroom facilities
Laura (LabLogic)	6.1.4.62SP1	Chromatography data collection and analysis software program used on HPLC, TLC and GC quality control equipment

### Radiolabeling

The ABY-025 precursor (Z_HER2:2891_-Cys-MMA-DOTA, 0.9 mg/mL in 0.1 M sodium acetate pH 5.3 filled in sterile class I plus vials) was manufactured by GMP standard solid-phase peptide synthesis, as described earlier (Feldwisch et al. 2010) and kindly provided for the study by Affibody AB. The amino acid sequence for ABY-025 was previously described by Ahlgren et al. (2010) Automated radiolabeling was performed using an Eckert & Ziegler Modular-Lab PharmTracer synthesis module. The reagent kit (EZ-102) and hardware kit (C4-GA-PEP, single-use cassette) for synthesis of ^68^Ga-peptides designed for Modular-Lab PharmTracer synthesizer was purchased from Eckert & Ziegler. The ^68^GaCl_3_ eluate was obtained from an Eckert & Ziegler 50 mCi GalliaPharm ^68^Ge/^68^Ga-generator. The synthesis method sequence for the Modular-Lab PharmTracer software (version 6.2) was developed in-house at the Karolinska Radiopharmacy department, Karolinska University Hospital. The developed method sequence is based on an original sequence provided by Eckert & Ziegler (68Ga-DOTA-Peptides, acetone free). Exact details on the synthesis method sequence can be read from the exemplified batch report in the Additional file 1. Additionally, exact details on the materials and chemicals used in this study are described in the Additional file 2: Table S1.

#### ^68^Ga-eluate preparation

To obtain the ^68^Ga-eluate, 5.0 mL of 0.1 N hydrochloric acid (HCl) (Eckert & Ziegler) was eluted through the generator into a sterile glass 15 mL vial (Huayi). The ^68^Ga-eluate obtained from the generator was measured in a dose calibrator (Capintec) to obtain an exact starting activity. The eluate was subsequently transferred to the synthesis hotcell in a lead shielded vial and then further to the synthesis unit via a syringe driven transfer operated by the Modular-Lab software. This procedure was chosen to disturb the clinically ongoing ^68^Ga-kit ([^68^Ga]Ga-DOTATOC and [^68^Ga]Ga-PSMA-11) preparation management as little as possible.

#### Synthesis of [^68^Ga]Ga-ABY-025

**Fig. 1:**
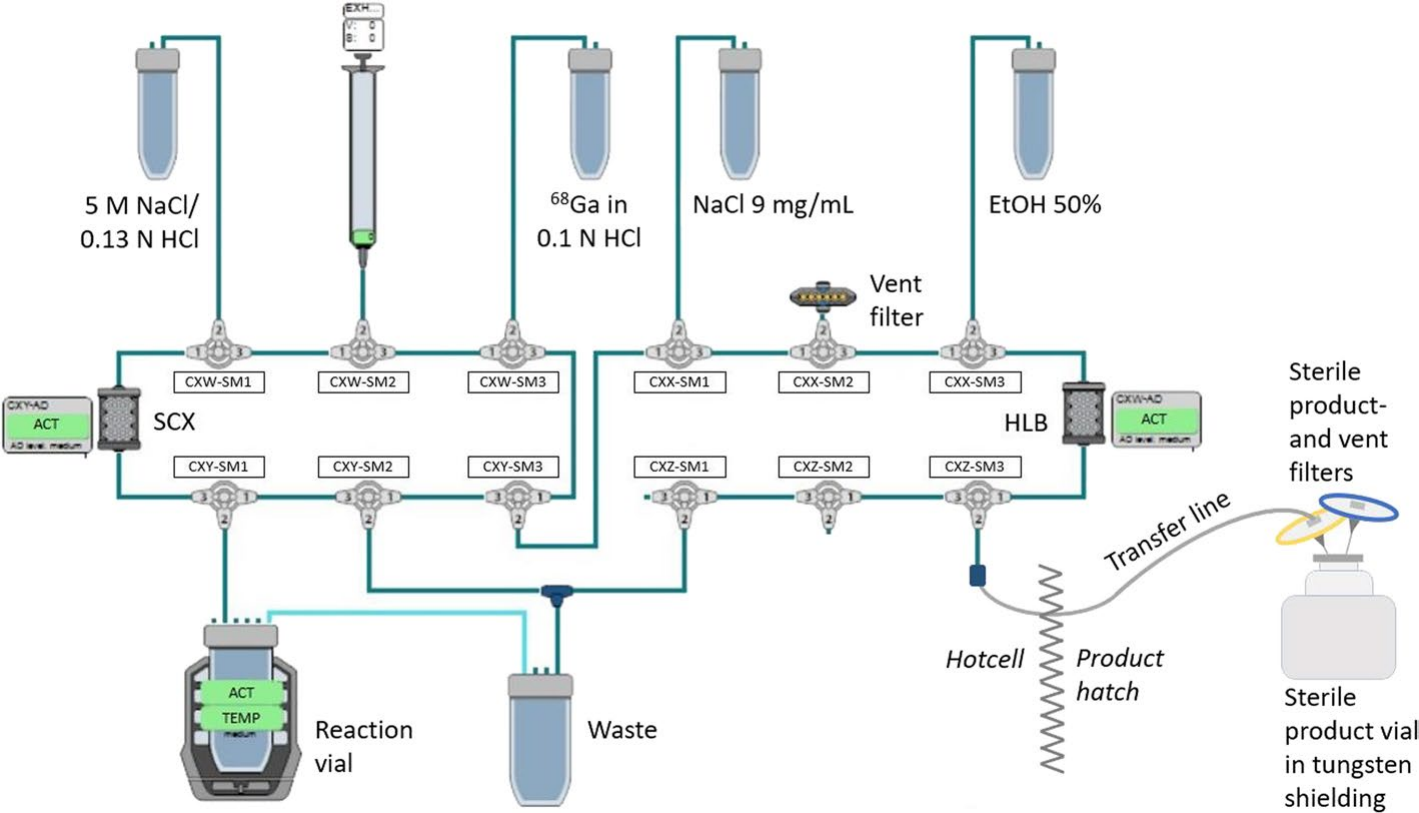
Schematic illustration of the radiosynthesis set-up, including cassette, transfer line, and product vial

**Fig. 2:**
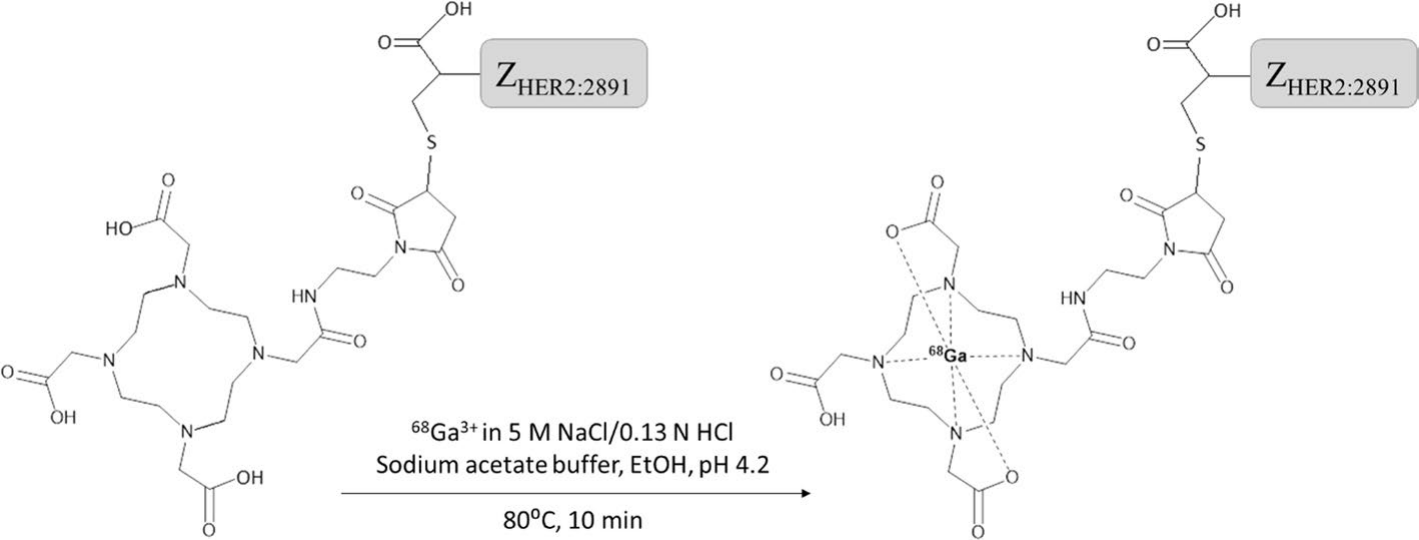
Molecular structure illustration and radiolabeling conditions of ABY-025

The buffer solution was prepared in the following way using the EZ-102 reagent kit (according to the Eckert & Ziegler provided “User Manual for synthesis [^68^Ga]-conjugated peptides with PharmTracer fractionation and pre-purifications by cation exchange”, section PSMA-11): 4.5 mL of the solution in vial 2a was transferred to the sodium acetate trihydrate vial (vial 2). From the buffer solution in vial 2, a volume of 0.4 mL was then transferred to the 6 mL mixing vial. To the mixing vial 1 mL (0.9 mg) of ABY-025 and 0.4 mL of 50% ethanol (vial 3 in the reagent kit) was also added. The final concentrations of components in the mixing vial were approximately 0.25 M sodium actetate buffer, 0.5 mg/mL ABY-025, and 11.1% ethanol. The buffer/peptide/ethanol solution was then finally transferred to the reaction vial of the cassette. The purification column, reversed phase solid phase extraction (SPE) cartridge (Oasis hydrophilic-lipophilic balanced (HLB) light cartridge (30 mg sorbent)) (Waters), was attached to the cassette instead of the C18 plus light cartridge (pre-mounted on the C4-GA-PEP cassette) and activated by the synthesis software using 50% ethanol and sodium chloride (NaCl) 9 mg/mL whilst the reaction solution was prepared by the operator. 3 mL of eluent solution (NaCl (5M)/HCl (0.13N) from vial 1 in the EZ-102 reagent kit) was also transferred to the intended vial of the synthesis cassette. The ^68^Ga was eluted from the generator and then trapped on a cationic exchange cartridge (SCX, pre-mounted on the C4-GA-PEP cassette, see Fig. 1) and eluted into the reaction vessel with 0.7 mL of eluent solution, a method inspired from Mueller et al. (2012). The final volume of the reaction mixture was 2.5 mL, pH 4.2. The labeling reaction mixture was heated to 80 °C for 10 min. After the end of the radiolabeling the crude product was diluted with 2 mL of NaCl 9 mg/mL and trapped on the SPE. The SPE was thereafter rinsed to waste using 4 mL of NaCl 9 mg/mL to remove any remaining free ^68^Ga ions in the system. The trapped product was then eluted from the SPE, using 1.2 mL of 50% ethanol, through a 0.22 µm/Ø 33 mm sterile filter (Millex-GV, Millipore) into the product vial. The product, [^68^Ga]Ga-ABY-025, was lastly diluted with NaCl 9 mg/mL to a final formulation volume of approximately 9.5 mL. Approximately 300 µL was withdrawn for the following QC analyses, plus 1 mL for the sterility analysis. A schematic illustration of the radiosynthesis set-up is shown in Fig. 1. A molecular structure illustration and radiolabeling conditions of ABY-025 is shown in Fig. 2.

#### Production method utility

In addition to the ongoing study, we extended this optimized synthesis method to another Affibody molecule, a ^68^Ga-labelled PDGFR-beta binding peptide for a planned clinical trial. The peptide is previously described in pre-clinical studies (Strand et al. 2014; Tolmachev et al. 2014). This aimed to validate the adaptability and versatility of the optimized synthesis method. Approximately 300 µL of PDGFR-beta Affibody molecule (concentration 1 mg/mL in water), was used in this synthesis following the same method as described above.

### Process validation

The process validation was conducted to provide documented evidence that the manufacturing process of [^68^Ga]Ga-ABY-025, when operated by trained personnel, was proper, robust and generates a final product with desired quality. The validation results are based on four batches, with three batches including the sterile filtration and one batch omitting the sterile filtration of the product, with one product vial per batch. The reason for excluding the final sterile filtration in one batch was to control for potential microbiological burden originating from the radiosynthesis equipment (a worst-case scenario). Full QC analyses were performed (see “Quality control” section) on all batches.

### Cleaning validation

A cleaning validation (CLV) was performed on the product transfer line (Tefzel® ETFE Tubing, GE Healthcare) between the synthesis module hotcell and the product hatch compartment. The CLV was based on three batches. Chemical analysis of any potential chemical residues remaining after transfer line cleaning (10 mL sterile water, 10 mL sterile 70% ethanol, flushing with helium gas for 8 min) by high performance liquid chromatography (HPLC) and test of pH on all CLV batches. Possible carry-over products included in the analysis were ABY-025, HCl, sodium acetate buffer, and possible unidentified impurities. Sampling was performed using 1 mL sterile water.

### Quality control

The analytical procedures used to control the drug product were thoroughly validated against the European Pharmacopoeia specifications prior to the process validation of 4 consecutive batches. The analytical procedures and acceptance limits are based on current published European Pharmacopoeia (Ph.Eur) monographs for ^68^Ga-labeled products, i.e. Ph.Eur monograph 01/2013:2482 for [^68^Ga]Ga-DOTATOC and Ph.Eur monograph 04/2021:3044 for [^68^Ga]Ga-PSMA-11. Product specifications are listed in Table 2.

**Table 2:** Product specifications for [^68^Ga]Ga-ABY-025

Test attributes	Product Specification
Radioactivity concentration	≥ 30 MBq/mL
Appearance	Clear and/or slightly yellow. Free of visible particles
pH	4.0–8.0
Product identity [^68^Ga]Ga-ABY-025	|Rt_RD_ − Rt_UV_| ≤ 60 s
Concentration of ABY-025 (principal peak on UV)	40–60 µg/mL
Radiochemical impurity [^68^Ga]-ions and/or unidentified radioactive impurities = B	≤ 3%
Total radiochemical purity[^68^Ga]Ga-ABY-025 RCP_Tot_ = (100–B) × T	≥ 91%
Chemical impurity (peaks except principal peak on UV)	≤ 10 µg/mL
Filter integrity	≥ 3.5 bar
Bacterial endotoxins	< 17.5 IU/mL
Ethanol content	< 80 mg/mL
Sterility	Sterile, 0 CFU
Radionuclidic identity, half-life ^68^Ga	62–74 min
Radiochemical stability 2 h EOS	RCP_Tot_ ≥ 91%

Rt_RD_ = retention time from radiodetector; Rt_UV_ = retention time from UV detector, EOS = end of synthesis, RCP_Tot_ = Total radiochemical purity; B = percentage of radioactivity due to impurity ^68^Ga-ions and/or unidentified radioactive impurities from iTLC analysis; T = proportion of the radioactivity due to [^68^Ga]Ga-ABY-025 in the HPLC analysis

**Appearance** The product is visually inspected for its clarity and the absence of visible particles after sufficient radioactive decay in order to avoid high radiation dose to the operator. This quality control is performed on validation and verification batches only.

**pH** Estimated by pH paper (VWR) or pH-meter (type 913, version 2.913.0210, Metrohm). This quality control is performed pre-release on all batches.

**Radiochemical identity** Radiochemical product identity is determined by comparison of a sample from the formulated [^68^Ga]Ga-ABY-025 product solution with a reference solution of ABY-025 pre-analyzed, using a HPLC system. The eluent is monitored by an ultraviolet (UV) detector and a radio detector placed in series. See Table 3 for instrument and method setup. This quality control is performed pre-release on all batches.

**Table 3:** Analytical HPLC method instrument setup

HPLC Agilent 1260 (Agilent/LabLogic)	Pump G7111B Manuel injector G1328C (50 µL) 6-column selection valve G1170A (optional) VWR detector GG7114A Radiodetector Flow-RAM • 1″ NaI PMT • Blue tubing 0.25 mm, wrapping 5 rounds Evaluation software Laura 6 (LabLogic)		
Column	Analytical column: Poroshell 120 EC-C18, 3 × 150 mm, 2.7 µm Guard Column: Poroshell 120 EC-C18 Fast guard, 3 × 5 mm, 2.7 µm		
Mobile phase	A: 0.1% trifluoroacetic acid in water B: acetonitrile C: 80% acetonitrile in water		
Gradient	Time (min)	Mobile phase composition	
	Initial	80% A	20% B
	1 min 30 s	80% A	20% B
	10 min	40% A	60% B
	11 min	40% A	60% B
	11 min 10 s	80% A	20% B
Run time	15 min		
Flow Rate	0.3 mL/min		
Column temperature	Room temperature		
Injection volume:	50 µL		
Detection	UV absorbance at 220 nm		

**Concentration of ABY-025** Peptide concentration is determined by comparison of a sample from the formulated [^68^Ga]Ga-ABY-025 product solution with a pre-set calibration curve of ABY-025, using a HPLC system. The eluent is monitored by an UV detector. See Table 3 for instrument and method setup. This quality control is performed pre-release on all batches.

**Fig. 3:**
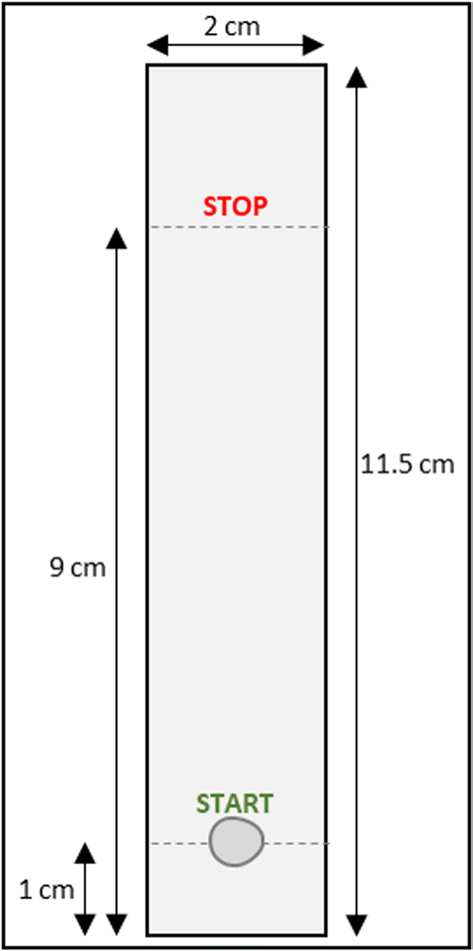
Schematic illustration for preparation and elution of iTLC plate

**Radiochemical purity, Impurity B** Impurity B, unbound ^68^Ga-ions, is determined by instant Thin Layer Chromatography (iTLC) using a radioactivity detector. See Table 4 and Fig. 3 for instrument and method setup. This quality control is performed pre-release on all batches.

**Table 4:** Analytical iTLC method instrument setup

Scan-RAM radio-TLC scanner (LabLogic)	Detector PS Plastic/PMT Evaluation software Laura 6 (LabLogic)
Stationary phase	Glass microfiber chromatography paper impregnated with a silica gel (SG) (Agilent)
Mobile phase	0.1 M citrate buffer, pH 5
Application volume	4 µL
Elution	Start from 1 cm, stop at 9 cm

**Total radiochemical purity** The total radiochemical purity (RCP_Tot_) is determined by HPLC and iTLC with radioactivity detection and is calculated as:

$${RCP}_{Tot}=\left(100-B\right) \times T$$

where B = Percentage of radioactivity due to impurity ^68^Ga^3+^ in iTLC analysis; T = proportion of the radioactivity due to [^68^Ga]Ga-ABY-025 in the HPLC analysis. See Table 3 and 4 for instrument and method setup. This quality control is performed pre-release on all batches.

**Chemical impurities** The chemical impurities of [^68^Ga]Ga-ABY-025 product solution was estimated from the UV peaks not corresponding to ABY-025 by HPLC analysis. See Table 3 for instrument and method setup. This quality control is performed pre-release on all batches.

**Filter integrity** Filter integrity was determined by a bubble point test, using a custom-made equipment (010105280602-A, DM Automation). This quality control is performed pre-release on all batches.

**Bacterial endotoxins** Bacterial endotoxins content is determined using the chromogenic kinetic methodology on Endosafe® Nextgen-PTS Kinetic Reader using Test Cartridge PTS2005F. The limit for the maximum endotoxin concentration of the product was set to correspond to a maximum product injection volume of 10 mL. This quality control is performed pre-release on all batches.

**Solvents** The concentration of ethanol is determined by gas chromatography (GC). This quality control is performed on all batches; the product may be released before completion of this test. See Table 5 for instrument and method setup.

**Table 5:** Analytical GC method instrument setup

Gas Chromatography Model 6850 (Agilent)	Flame ionization detector (FID) Evaluation software Laura 6 (LabLogic)
Column	Res-Solv (30 m × 0.53 mm id × 1.0 µm film), autoinjector
Split ratio	1:78
Gases	Helium (carrier gas), hydrogen and synthetic air
Injection volume:	2 µL
Inlet temperature	250 °C
Inlet pressure	2.76 PSI
Temperature gradient	35 °C isotherm for 4 min after injection, ramp to 100 °C at 80 °C/min, hold at 100 °C for 30 s, ramp to 220 °C at 80 °C/min, hold at 220 °C for 30 s, and cool to 35 °C”

**Sterility** Sterility is determined by direct inoculation according to the Ph.Eur. This quality control is performed post-release on validation batches and on every 10th clinical batch or every 3rd month, whichever occurs first.

**Radionuclidic identity** Radionuclidic identity is confirmed by comparing the half-life of the product with that of ^68^Ga (62–74 min). The half-life is calculated by repeated measurements of product radioactivity using a dose calibrator (CRC-55TR, Capintec). This measurement is performed on the validation and verification batches only.

**Radionuclidic purity** We do not analyze the radionuclidic purity on the product. Instead, we analyze for radionuclidic purity of the ^68^Ga eluate as part of the release of the generators, using an HPGe-MCA detector (Canberra, Mirion) with Cryo-Cycle 2TM Hybrid Cryostat.

### Validation of analytical methods

The acceptance limits and parameters for the validation of analytical methods are summarized in Table 6.

**Table 6:** Acceptance limits and parameters for the validation of analytical methods (to assure that the analytical methods can properly determine specifications set in Table 2)

HPLC	Specificity	Sample matrix spiked with ABY-025: Area of ABY-025 peak in spiked matrix differ maximum ± 20% and that no peaks ≥ 10% of the area of 50 µg/mL ABY-025 in the range 6–10 min in UV Sample matrix spiked with ^68^Ga: ^68^Ga should elute only in the void peak in the RD Difference in Rt between [^68^Ga]Ga-ABY-025 (radio chromatogram) and Rt ABY-025 (UV-chromatogram of SST) ≤ 60 s
	Linearity of chemical purity for ABY-025	Peak area in at least 5 levels in the range 30–100 µg/mL. Linear regression should give a r^2^ ≥ 0.99 Carry over: The ABY-025 peak in blank after the 100 µg/mL sample should not exceed 20% of peak in previous injection
	Linearity of radiochemical purity for [^68^Ga]Ga-ABY-025	Linearity is tested for samples from 10 MBq/mL to 150% of normal product activity (75 MBq/mL) Linear range for the method are defined as the activity range where %ROI [^68^Ga]Ga-ABY-025 differs ≤ 0.5%ROI for the average (of samples within range)
	Repeatability	The repeatability is tested in the range 30–100 µg/mL ABY-025. The relative response, as compared with the SST should show an RSD% ≤ 10% on each concentration level (3 injections) and for all injections (9 injections) within the range 30–100 µg/mL (3 concentrations / 3 replicates). Relative response = (Area/Conc.)_Sample_ / (Area/Conc.)_SST/Ref_
	Intermediate precision	On 3 occasions 3 triplicates on 3 concentrations are performed. Different days or analysts, at least two columns from different lots. The relative response, as compared with the SST should show an RSD% ≤ 10% on each concentration level (9 injections) and for each analysist (9 injections)
	Accuracy	Accuracy can be inferred once precision, linearity and specificity has been established. Accuracy should be shown in the range 30–100 µg/mL
GC	Linearity	R^2^ ≥ 0.99; Slope, y-intercept and residual sum of square must be given. A minimum of 5 concentrations must be used LOD and LOD: Calculation based the standard deviation of the response and the slope (based on calibration curve)
	Repeatability	RSD% ≤ 10% for a minimum of 9 determinations over 3 concentrations within the range (e.g. 3 concentrations /3 replicates from Accuracy)
	Intermediate precision	RSD% ≤ 10%; for a minimum of 9 determinations over 3 concentrations within the range (e.g. 3 concentrations/3 replicates from Accuracy) performed a different day or by a different analyst
	Accuracy	Diff% ≤ 10% between calculated and actual concentrations for 3 concentrations within the range using a minimum of 3 replicate determinations from each concentration
TLC	Specificity	i.e. its ability to determine free ^68^Ga and/or unidentified radioactive impurities. The following criteria must be met: • The peak corresponding to free ^68^Ga and/or unidentified radioactive impurities must be completely separated from the peak corresponding to [^68^Ga]Ga-ABY-025 • The radioactivity of free ^68^Ga and/or unidentified radioactive impurities must be ≤ 3% of the total radioactivity to be released • Retardation factors (Rf) ~ 0–0.1 for free ^68^Ga and/or unidentified radioactive impurities Rf ~ 0.2–0.6 and Rf > 0.6 for [^68^Ga]Ga-ABY-025
Endotoxins	Optimal dilution factor	Interference screen on worst case simulation of [^68^Ga]Ga-ABY-025 formulation using maximum allowed ethanol concentration The optimal dilution factor is the one that yields a spike recovery close 100% (related to the known endotoxin level in the cartridge)
Sterility	MST	MST at the external contractor according to their certified protocol

RD = Radiodetector, ROI = Region of interest, Rt = Retention time, SST = Suitability test

## Results

### Process validation results of [^68^Ga]Ga-ABY-025

An automated radiolabeling method for [^68^Ga]Ga-ABY-025 was successfully developed. With a decay corrected radiochemical yield of 44 ± 2%, a radiochemical purity of 98 ± 1% at end of synthesis (EOS), and a radiochemical stability of 98 ± 1% 2 h EOS (n = 4), the synthesis method was found highly reproducible. During radiolabeling development of the product, no radiolysis was observed, therefore no further stabilization (for example with use of ascorbate) was needed. Early attempts using the previously described buffer preparations as for [^68^Ga]Ga-DOTATOC and [^68^Ga]Ga-FAPI-46 (Jussing et al. 2021) did not result in desired yields nor product quality. Also, the SPE purification cartridge pre-mounted on the C4-GA-PEP cassette (C18 plus light), was changed to an HLB cartridge for successful trapping and releasing of the [^68^Ga]Ga-ABY-025 product in the last step of the radiosynthesis procedure. The change of SPE was performed as recommended by previously published data on the purification strategy for crude radiotracers based on Affibody molecules (Velikyan et al. 2016; Jussing et al. 2020). The finally chosen pH, temperature, and reaction heating time were optimized in regards of radiolabeling efficiency, and were found tolerated by the ABY-025 peptide. Higher or lower reaction pH values significantly lowered the radiochemical yields, which was interpreted to be caused by the formation of ^68^Ga-colloids or inadequate radiolabeling. Higher reaction temperatures and increased reaction time were observed to degrade the ABY-025 peptide, thus lowering the radiochemical/chemical purity. Shorter reaction times and lower reaction temperatures did not result in desired yields. It was discovered that approximately 50% of the ABY-025 peptide was lost from addition in the reaction vial to what was present in the final product (compare the 0.9 mg addition in the reaction vial to the chemical concentration of ABY-025 presented in Table 7). Aiming for a final product containing approximately 0.5 mg of peptide [an earlier determined optimal dosage published by Sorensen et al. (2016)], adding 0.9 mg of peptide into the reaction vial was found suitable. With this approach an addition of peptide in the formulation step of the product vial, as previously described (Velikyan et al. 2019) could be avoided in current study.

**Table 7:** Batch analysis for 4 validation batches of [^68^Ga]Ga-ABY-025

Test attributes	Product Specification	Batch 1	Batch 2	Batch 3	Batch 4	Statistical data (n = 4)
Activity EOS (after withdrawal of QC- and sterility samples)	≥ 300 MBq	363 MBq	385 MBq	380 MBq	392 MBq	380 ± 12 MBq
Volume (after withdrawal of QC- and sterility samples)	≤ 10 mL	7.8 mL	8.0 mL	8.0 mL	8.8 mL^1^	8.2 ± 0.4 mL
Activity concentration	≥ 30 MBq/mL	47 MBq/mL	48 MBq/mL	48 MBq/mL	45 MBq/mL	47 ± 1 MBq/mL
Radiochemical yield (decay-corrected)^2^	N/A	41%	46%	43%	46%	44 ± 2%
Appearance	Clear and/or slightly yellow. Free of visible particles	Complies	Complies	Complies	Complies	Complies
pH	4.0–8.0	5.0	5.0	5.0	5.0	5.0 ± 0.0
Product identity [^68^Ga]Ga-ABY-025	|Rt_RD_ − Rt_UV_|≤ 60 s	11 s	7 s	12 s	13 s	11 ± 3 s
Concentration of ABY-025 (principal peak on UV)	40–60 µg/mL	50 µg/mL	50 µg/mL	60 µg/mL	60 µg/mL	55 ± 6 µg/mL
Chemical impurity (peaks except principal peak on UV)	≤ 10 µg/mL	< 4 µg/mL	< 4 µg/mL	< 4 µg/mL	< 4 µg/mL	< 4 µg/mL
Radiochemical impurity [^68^Ga]-ions and/or unidentified radioactive impurities = B	≤ 3%	1%	0%	1%	2%	1 ± 1%
Total radiochemical purity[^68^Ga]Ga-ABY-025 RCP_Tot_ = (100 − B) × T	≥ 91%	99%	98%	98%	98%	98 ± 1%
Filter integrity^3^	≥ 3.5 bar	4.3	4.1	4.2	N/A	4.2 ± 0.1 bar
Bacterial endotoxins	< 17.5 IU/mL	< 5 IU/mL	< 5 IU/mL	< 5 IU/mL	< 5 IU/mL	< 5 IU/mL
Ethanol content	< 80 mg/mL	45 mg/mL	45 mg/mL	48 mg/mL	44 mg/mL	46 ± 2 mg/mL
Sterility	Sterile, 0 CFU	Sterile	Sterile	Sterile	Sterile	Sterile
Radionuclidic identity Half-life ^68^Ga	62–74 min	67 min	69 min	67 min	69 min	68 ± 1 min
Radiochemical stability 2 h EOS	RCP_Tot_ ≥ 91%	98%	98%	98%	97%	98 ± 1%

Rt_RD_ = retention time from radiodetector; Rt_UV_ = retention time from UV detector, EOS = end of synthesis, RCP_Tot_ = total radiochemical purity; B = percentage of radioactivity due to impurity ^68^Ga-ions and/or unidentified radioactive impurities from iTLC analysis; T = proportion of the radioactivity due to [^68^Ga]Ga-ABY-025 in the HPLC analysis; ^1^Larger volume since no product was absorbed by the sterile filter; ^2^28 min between measurement of ^68^Ga-eluate and the final product; ^3^n = 3 since sterile filtration was excluded for Batch 4

[^68^Ga]Ga-ABY-025, produced according to the method described in the method section, has been validated in four consecutive batches under normal operating conditions. A microbiological worst-case challenge (i.e. a batch where the sterile filter is intentionally excluded), was performed on one of the batches. Results obtained for these validation runs, are presented in Table 7. Statistical data was calculated by using the mean (= AVERAGE) and the standard deviation based on a sample (= STDEV.S) formulas in Excel (Microsoft® Excel® for Microsoft 365 MSO (Version 2208).

#### Production method utility

Results from the attempt of applying the same production method (n = 4) on another ^68^Ga-labeled radiotracer based on Affibody molecules (PDGFR-beta targeting) were successful. With a decay-corrected radiochemical yield of 60 ± 2%, a radiochemical purity of 98 ± 0% at EOS, the synthesis method was found highly reproducible also for this radiotracer.

### Cleaning validation of product transfer line

The CLV results were consistent and provided sufficient evidence that the cleaning procedure for the transfer line between the synthesis module and the final product vial in the product hatch successfully removed potential chemical residues after production of [^68^Ga]Ga-ABY-025. Possible carry-over products included in the analysis (ABY-025, HCl, sodium acetate buffer, and possible unidentified impurities) were not detected by HPLC or pH measurement.

### Product quality control analyses

**Fig. 4:**
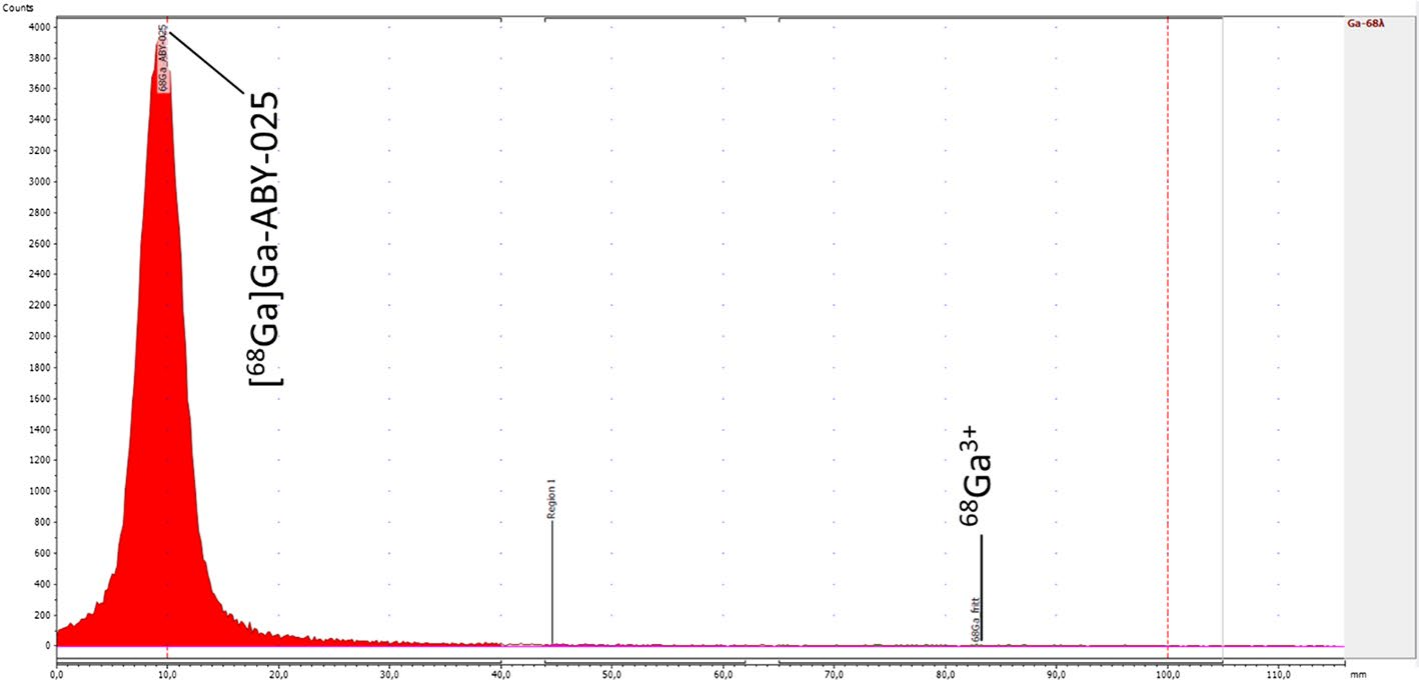
Example of chromatogram obtained from the iTLC analysis, determining the radiochemical purity. Counts on the y-axis, minutes on the x-axis

**Fig. 5:**
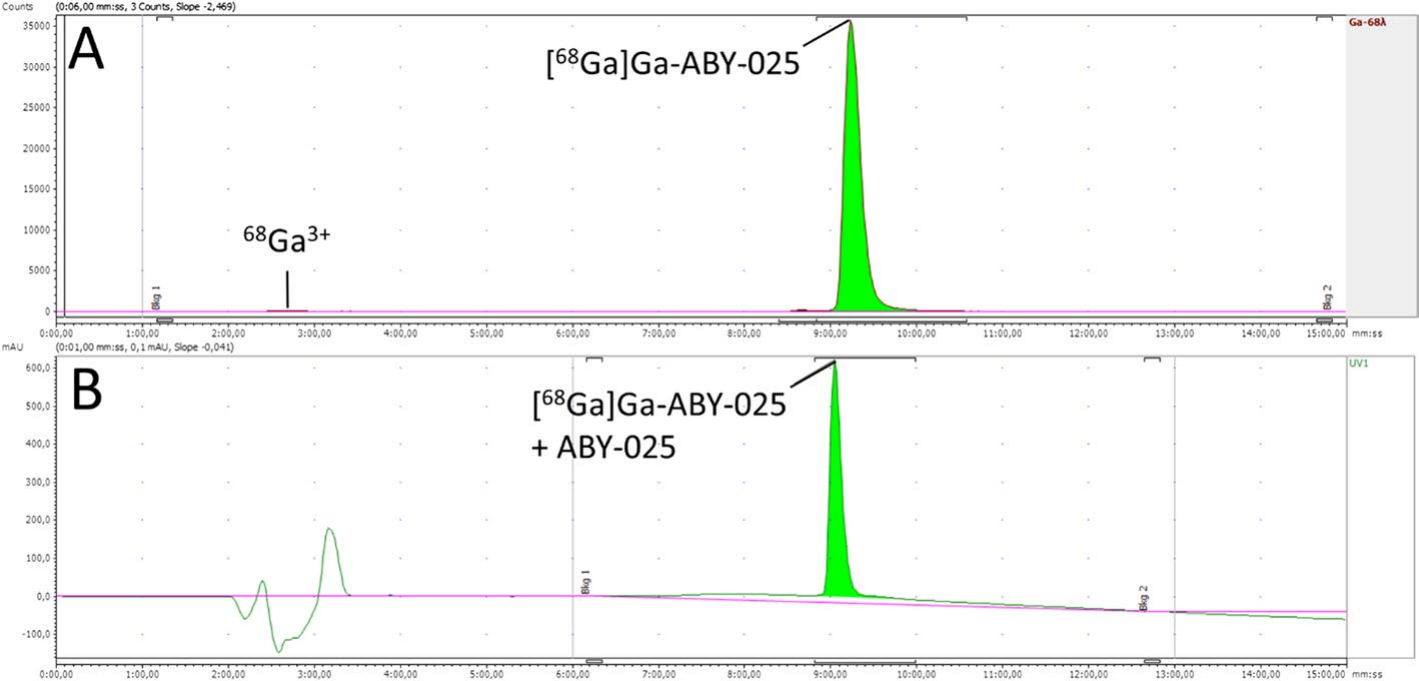
Example of chromatograms obtained from the HPLC analysis, determining the radiochemical purity (**A**) and identity (**B**). For **A**: counts on the y-axis, minutes on the x-axis. For **B**: milli-absorbance units on the y-axis, minutes on the x-axis

**Fig. 6:**
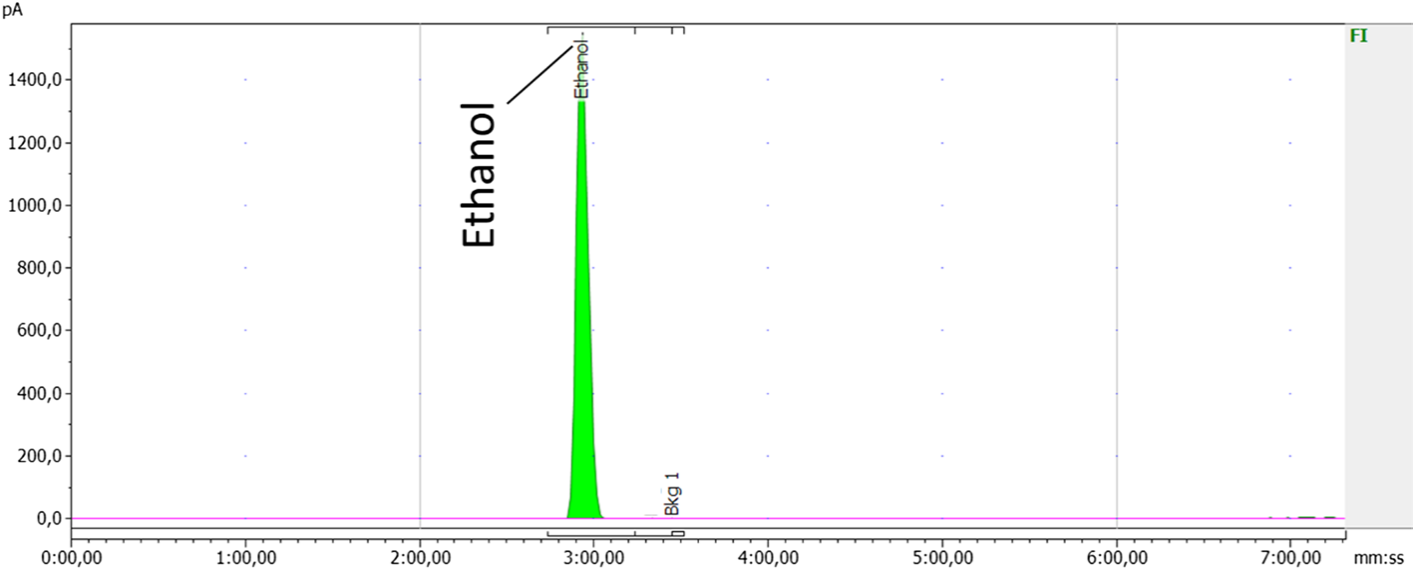
Example of chromatogram obtained from the GC analysis, determining the ethanol content. Current units on the y-axis, minutes on the x-axis

The QC methods were confirmed, through the validation of analytical methods, to be reliable and suitable for their intended use. Results from the quality control analyses for all product validation batches are presented in Table 7. Examples of chromatograms obtained for the iTLC, HPLC, and GC analyses are shown in Figs. 4, 5 and 6, respectively.

## Discussion

Breast cancer patients that would benefit from effective treatment with HER2 targeted drugs, such as trastuzumab, has until recently been considered to be those showing a high HER2 tumor expression. In a clinical trial (NCT01858116) [^68^Ga]Ga-ABY-025 was used as a HER2 specific radiotracer for PET imaging, for binary categorization of the lesions, e.g. either HER2 positive or negative, showed promising results (Sorensen et al. 2016). Recent clinical trial results indicate that also breast cancer patients with HER2-low expressing cancers would benefit from trastuzumab (together with deruxtecan (T-Dxd)) treatment, with significantly improved progression-free survival and overall survival. Promising results also for this cohort of HER2-low expressing cancers has gained attention and is predicted to change the treatment regime for breast cancer, adding HER2-low as another category for treatment with targeted drugs (Seban et al. 2023; Zhang et al. 2022). A new clinical trial (NCT05619016) aims to improve the selection of patients who would benefit from effective treatment with HER2 targeted drugs. HER2 expressing metastatic solid tumors such as breast cancer and gastro-esophageal adenocarcinoma will be evaluated with HER2-PET and [^68^Ga]Ga-ABY-025 in this trial. Imaging data will be confirmed and compared to HER2 expression in tumor tissue derived from biopsies. A pilot study in cohort of metastatic breast cancer with HER2-low expression is finalized (manuscript under submission). Results are very encouraging and could be expanded to a great number of other HER2-expression tumors. The potential number of required HER2-PET investigation could rise tenfold making the scope of this work very actual.

In the previous study using [^68^Ga]Ga-ABY-025 (Sorensen et al. 2016) the radiotracer has been radiolabeled either by a completely manual- or automated method, both including a fractionation of the ^68^Ga-eluate and an addition of “cold” ABY-025 as a final step. For the clinical trial NCT05619016 an alternative automatic radiolabeling cGMP-compliant method for [^68^Ga]Ga-ABY-025 was developed. Optimizations were performed to replace the fractionation of the ^68^Ga-eluate to a pre-concentration procedure, and to remove the necessity of adding “cold” ABY-025 in the final step. The radiolabeling is performed on a commercial synthesis module (Modular-Lab PharmTracer, Eckert & Ziegler), used worldwide for production of ^68^Ga-based radiopharmaceuticals. The synthesis cassette (C4-GA-PEP, Eckert & Ziegler) and reagent kit (EZ-102, Eckert & Ziegler) are off-the-shelf products. The radiolabeling method, along with following QC methods, are clearly explained to ease reproducibility and possible future tech transfer.

To simplify the method even further, the possibility to use the already mounted purification cartridge (C18 plus light) on the synthesis cassette, not requiring a replacement before start of synthesis, could be investigated. In current study the decision to not adjust the proven successful purification procedure was made, compared to already published production methods for [^68^Ga]Ga-ABY-025 (Velikyan et al. 2016, 2019).

In accordance with relevant guidelines for radiopharmaceutical manufacturing, as well as pharmaceutical manufacturing in general (EudraLex vol.4, Annex 1), manual manipulations to the process should be kept to a minimum. This is of course to protect the product from microbial contamination, as any additional manual process steps adds on an extra risk to interfere with the products´ final sterility. The risk of human/operator bound variations between batches, errors, or in the worst-case complete failures, is obviously also connected to the number of manual process steps added to the manufacturing process. A fully automated and robust radiosynthesis method, as described in current study, was carefully developed to meet these cGMP standards.

Less important for patient safety aspects, but more important for the operator, hands-on interactions will increase the operators’ radiation burden in the manufacturing of radiopharmaceuticals. The principle of “As Low As Reasonably Achievable” (ALARA) clarifies even further that minimizing the number of process steps, involving radioactive exposure, is desirable (Frane and Bitterman 2023). This is achieved in current study by performing the radiosynthesis inside the shielding of a hotcell, and then transferring the product to an adjacent compartment, a product hatch. The product can then be collected without exposure to the residual radioactivity in the synthesis equipment.

Previous attempts with pre-concentration of ^68^Ga-eluate and subsequent radiolabeling failed due to that i.e. ABY-028 couldn’t handle the high NaCl concentrations in the elution step (5M) (Jussing et al. 2020). However, effort was made to succeed with this optimized feature to the radiosynthesis. The key was found to concern an adjustment of the reaction buffer solution. The result is a refined radiopharmaceutical production method spared from manual interactions, subsequent start of synthesis. With this optimization utilization of the full ^68^Ge/^68^Ga generator eluate was made possible, with less radioactivity going to waste. It should be noted that the procedure chosen in current study, to first elute the ^68^Ga into a vial for transport into the hotcell before connection to the synthesis module, could easily be replaced by a direct connection of the ^68^Ge/^68^Ga-generator. The only reason for the chosen procedure in this study was to disturb the clinically ongoing ^68^Ga-kit ([^68^Ga]Ga-DOTATOC and [^68^Ga]Ga-PSMA-11) preparation management as little as possible, thus not transferring the ^68^Ge/^68^Ga-generator in and out from the hotcell. The current automated method is in line and comparable with most commercial ^68^Ga-labeling methods using the same synthesis module, with harmonization and ease as consequences.

Additional to the presented optimization of the production method for [^68^Ga]Ga-ABY-025 of this study, it was further proved that the method also can be applied on ^68^Ga-labeling other radiotracers based on Affibody molecules. This indicates that the production method might be applied on ^68^Ga-labeling radiotracers based on Affibody molecules, or other similar molecules, in general.

Due to the successful introduction of the ^68^Ga-eluate pre-concentration step in the radiosynthesis method of [^68^Ga]Ga-ABY-025 it is likely to believe that the use of other sources of ^68^Ga is achievable, such as cyclotron produced ^68^Ga (Pharmacopoeia 2020) or even the approach to combine several ^68^Ge/^68^Ga-generator eluates to increase the radiolabeling starting activity. This also further emphasizes the utility of an automated synthesis and a radioactivity exposure safe environment to avoid unnecessary radiation burden for production personnel. We have earlier reported a solid-target production of ^68^Ga that could be incorporated into similar radiopharmaceutical productions, resulting in high-yield radiolabeling and a tenfold increase in molar activity (A_m_) (Jussing et al. 2021; Siikanen et al. 2021). Molecular diagnostics performed with the radiotracer in focus of current study, [^68^Ga]Ga-ABY-025, does not benefit from a high A_m_, but high A_m_ on ^68^Ga-labeled radiotracers might be of interest for other radiotracers based on Affibody molecules. Continuous studies are needed to investigate if this would be beneficial in pre-clinical or clinical diagnostics. The current study indicate that this can be investigated further.

## Conclusion

An optimized fully automated method for the radiosynthesis of [^68^Ga]Ga-ABY-025 was developed. The improvements consist of a pre-concentration of the ^68^Ga-eluate (to be able to utilize the full potential of the ^68^Ge/^68^Ga generator radioactivity output, i.e. less radioactivity going to waste) and the elimination of a peptide addition step to the final product vial. Moreover, the results from applying this method to another similar peptide offers compelling evidence of its broader applicability. This suggests that our method can be robustly employed in ^68^Ga-labeling of radiotracers based on Affibody molecules or other similar molecules.

### Supplementary Information

**Additional file 1.** Exemplified batch report.

**Additional file 2. Table S1.** Complete list of consumables and chemicals used.

## Data Availability

The datasets used and/or analysed during the current study are available from the corresponding author on reasonable request. Requests for ABY-025 should be made to Affibody AB.

## Abbreviations

ABY-025: Z_HER2:2891_-Cys-MMA-DOTA
A_m_: Molar activity (GBq/μmol)
cGMP: Current good manufacturing practice
CoA: Certificate of analysis
CTA: Clinical trial authorization
EOS: End of synthesis
GC: Gas chromatography
HCl: Hydrochloric acid
HER2: Human epidermal growth factor receptor 2
HLB: Hydrophilic-lipophilic balanced
HPLC: High performance liquid chromatography
IMPD: Investigational medicinal product dossier
iTLC: Instant thin-layer chromatography
MMA-DOTA: 1,4,7,10-Tetraaza cyclodecane-1,4,7-tris–acetic acid-10-maleimidoethylacetamide
MST: Method suitability test
NaCl: Sodium chloride
NaI PMT: Sodium iodide photomultiplier tube
PDGFR-beta Affibody molecule: Platelet-derived growth factor receptor-β Affibody molecule
PET: Positron emission tomography
RD: Radio-detector
ROI: Region of interest
Rt: Retention time
SPE: Solid phase extraction
SPECT: Single-photon emission computed tomography
SST: Suitability test
