# Supplementary material for: Optimized, automated and cGMP-compliant synthesis of the HER2 targeting [68Ga]Ga-ABY-025 tracer
Source: EJNMMI Radiopharm Chem. 2023 Nov 22;8:41. doi: 10.1186/s41181-023-00226-y (PMC10665286; doi:10.1186/s41181-023-00226-y)

Supplementary material: Exemplified Batch Report

Optimized, automated and cGMP-compliant synthesis of the HER2 targeting [^{68}Ga]Ga-ABY-025 tracer

Emma Jussing[1,2*], Mélodie Ferrat[1,2], Mohammad M Moein[1,2], Henrik Alfredéen[1,2], Tetyana Tegnebratt[1], Klas Bratteby[1,2], Erik Samén[1,2], Joachim Feldwish[4], Renske Altena[2,3], Rimma Axelsson[5,6], Thuy A Tran[1,2]

1. Department of Radiopharmacy, Karolinska University Hospital, SE-171 76 Stockholm, Sweden
2. Department of Oncology and Pathology, Karolinska Institutet, SE-171 77 Stockholm, Sweden
3. Karolinska Comprehensive Cancer Center, Karolinska University Hospital, SE-171 77 Stockholm, Sweden
4. Affibody AB, SE-171 65, Solna, Sweden
5. Department of Medical Radiation Physics and Nuclear Medicine, Karolinska University Hospital, SE-171 76 Stockholm, Sweden
6. Department of Molecular Medicine and Surgery, Karolinska Institutet, SE-171 77 Stockholm, Sweden

*Corresponding author: Emma Jussing, Ph.D., emma.jussing@ki.se

Batch Report

Device:	MTCYKL27	Procedure:	68Ga-DOTA-Peptides, acetone free
Batch:	ABY025-230629-1		
Started:	2023-06-29 11:53:35	Half life:	68 min
		Nuclide:	68Ga
Duration of synthesis:	00:22:44		
User-ID:	klbr		
Project path:	C:\PROGRAM FILES (X86)\EUROTOPE\ML62\PROJECTS\68GA-ABY-025-SCX-V1.0\68GA-ABY-025-SCX-V1.0.MDL		
Project version:	0.0.3		

Preparation steps:

1	Cassette mounted?	Ok
2	Waste vial connected?	Ok
3	NaCl (0.9 %) vial on left spike?	Ok
4	ethanol (50 %) vial on right spike?	Ok
5	Connect 68Ga eluate to cassette	Ok
6	Connect the transfer line ABY-025 to the cassette	Ok
7	Connect sterile vented product vial to the product line ABY-025 in the product hatch	Ok
8	Add 3 mL eluent SCX into eluent vial	Ok
9	Mix 0.4 mL buffer + 0.4 mL EtOH 50 % + 2 vials of ABY-025 precursor	Ok
10	Transfer reaction mixture into reaction vial	Ok

In Start batch dialog entered values:

68Ga labelling time (reaction time)	600
pre-heating reaction vial (pre-heat HRM)	50
labelling temperature (reaction temp HRM)	80
volume 0.1N HCl eluting generator (generator elution volume)	6000

Audit trail of process:

Timestamp	Object ID	Object Name	Process Step	Comment
2023-06-29 11:53:35	PCtrl	Flow Control	Batch control: start batch	
2023-06-29 11:53:36	PCtrl	Flow Control	Batch control: start audit trail	
2023-06-29 11:53:36	EXC-HR	EXC-HR	normal mode, Wait 200 ms Tsp(var.): 50 C	
2023-06-29 11:53:36	Set string	Set string	String: process_step = elute generator (const.)	
2023-06-29 11:53:36	CXX-SM2	CXX-SM2	Valve switched to direction 1-2	
2023-06-29 11:53:36	CXW-SM3	CXW-SM3	Valve switched to direction 1-2	
2023-06-29 11:53:36	CXW-SM2	CXW-SM2	Valve switched to direction 2-3	
2023-06-29 11:56:40	EXH-SY	EXH-SY	Syringe starts, volume(var.): 6000 ul, speed(var.): 33 ul/s	
2023-06-29 11:56:45	timer_1	Timer 1	Wait 5.00 s	
2023-06-29 11:56:45	CXW-SM3	CXW-SM3	Valve switched to direction 1-3	
2023-06-29 11:56:45	CXY-SM3	CXY-SM3	Valve switched to direction 1-2	
2023-06-29 11:56:50	EXH-SY	EXH-SY	Syringe starts, volume: 2000 ul, speed: 500 ul/s	
2023-06-29 11:56:50	CXY-SM2	CXY-SM2	Valve switched to direction 2-3	
2023-06-29 11:56:50	CXW-SM2	CXW-SM2	Valve switched to direction 1-2	
2023-06-29 11:56:50	Set string	Set string	String: process_step = 68Ga to SCX (const.)	
2023-06-29 11:57:51	EXH-SY	EXH-SY	Syringe starts, volume(var.): -6000 ul, speed(var.): 100 ul/s	
2023-06-29 11:58:04	EXH-SY	EXH-SY	Syringe starts, volume: -2000 ul, speed: 150 ul/s	
2023-06-29 11:58:04	CXX-SM2	CXX-SM2	Valve switched to direction 1-2	
2023-06-29 11:58:04	CXX-SM1	CXX-SM1	Valve switched to direction 1-3	
2023-06-29 11:58:04	CXY-SM3	CXY-SM3	Valve switched to direction 1-2	
2023-06-29 11:58:04	CXW-SM3	CXW-SM3	Valve switched to direction 1-3	
2023-06-29 11:58:05	CXW-SM2	CXW-SM2	Valve switched to direction 2-3	
2023-06-29 11:58:15	EXH-SY	EXH-SY	Syringe starts, volume: 5000 ul, speed: 500 ul/s	
2023-06-29 11:58:17	timer_1	Timer 1	Wait 2.00 s	
2023-06-29 11:58:17	CXW-SM2	CXW-SM2	Valve switched to direction 1-2	
2023-06-29 11:58:17	Set string	Set string	String: process_step = dry SCX (const.)	
2023-06-29 11:58:27	EXH-SY	EXH-SY	Syringe starts, volume: -5000 ul, speed: 500 ul/s	
2023-06-29 11:58:28	EXC-HR	EXC-HR	normal mode, Wait 200 ms Tsp(var.): 80 C	
2023-06-29 11:58:28	Set string	Set string	String: process_step = elute SCX (const.)	
2023-06-29 11:58:28	CXX-SM2	CXX-SM2	Valve switched to direction 1-2	
2023-06-29 11:58:28	CXX-SM1	CXX-SM1	Valve switched to direction 1-3	
2023-06-29 11:58:28	CXY-SM3	CXY-SM3	Valve switched to direction 1-2	

Batch Report (cont.)

Timestamp	Object ID	Object Name	Process Step	Comment
2023-06-29 11:58:28	CXW-SM3	CXW-SM3	Valve switched to direction 1-3	
2023-06-29 11:58:28	CXW-SM2	CXW-SM2	Valve switched to direction 2-3	
2023-06-29 11:58:34	EXH-SY	EXH-SY	Syringe starts, volume: 3000 ul, speed: 500 ul/s	
2023-06-29 11:58:36	timer_1	Timer 1	Wait 2.00 s	
2023-06-29 11:58:36	CXW-SM1	CXW-SM1	Valve switched to direction 2-3	
2023-06-29 11:58:36	CXW-SM2	CXW-SM2	Valve switched to direction 1-2	
2023-06-29 11:58:44	EXH-SY	EXH-SY	Syringe starts, volume: 2500 ul, speed: 400 ul/s	
2023-06-29 11:58:46	timer_1	Timer 1	Wait 2.00 s	
2023-06-29 11:58:46	CXY-SM1	CXY-SM1	Valve switched to direction 2-3	
2023-06-29 11:58:46	CXW-SM1	CXW-SM1	Valve switched to direction 1-3	
2023-06-29 11:58:46	EXC-HR	EXC-HR	normal mode, Wait 200 ms Tsp(var.): 80 C	
2023-06-29 11:58:46	Set string	Set string	String: process_step = eluent to SCX (const.)	
2023-06-29 11:58:54	EXH-SY	EXH-SY	Syringe starts, volume: -700 ul, speed: 100 ul/s	
2023-06-29 11:59:14	timer_1	Timer 1	Wait 20.00 s	
2023-06-29 11:59:14	CXW-SM1	CXW-SM1	Valve switched to direction 2-3	
2023-06-29 11:59:22	EXH-SY	EXH-SY	Syringe starts, volume: -2100 ul, speed: 300 ul/s	
2023-06-29 11:59:22	CXW-SM1	CXW-SM1	Valve switched to direction 1-3	
2023-06-29 11:59:22	EXC-HR	EXC-HR	normal mode, Wait 200 ms Tsp(var.): 80 C	
2023-06-29 11:59:22	Set string	Set string	String: process_step = wet SCX (const.)	
2023-06-29 11:59:25	EXH-SY	EXH-SY	Syringe starts, volume: -200 ul, speed: 100 ul/s	
2023-06-29 11:59:30	timer_1	Timer 1	Wait 5.00 s	
2023-06-29 11:59:30	Set string	Set string	String: process_step = 68Ga transfer (const.)	
2023-06-29 11:59:43	EXH-SY	EXH-SY	Syringe starts, volume: -2500 ul, speed: 200 ul/s	
2023-06-29 11:59:43	EXC-HR	EXC-HR	normal mode, Wait 200 ms Tsp(var.): 80 C	
2023-06-29 11:59:43	CXW-SM2	CXW-SM2	Valve switched to direction 2-3	
2023-06-29 11:59:43	Set string	Set string	String: process_step = dry SCX (const.)	
2023-06-29 11:59:54	EXH-SY	EXH-SY	Syringe starts, volume: 5000 ul, speed: 500 ul/s	
2023-06-29 11:59:56	timer_1	Timer 1	Wait 2.00 s	
2023-06-29 11:59:56	CXW-SM2	CXW-SM2	Valve switched to direction 1-2	
2023-06-29 12:00:06	EXH-SY	EXH-SY	Syringe starts, volume: -5000 ul, speed: 500 ul/s	
2023-06-29 12:00:06	CXY-SM1	CXY-SM1	Valve switched to direction 1-3	
2023-06-29 12:00:06	Set string	Set string	String: process_step = reaction time (const.)	
2023-06-29 12:00:06	Set string	Set string	String: process_Step_2 = (const.)	
2023-06-29 12:00:06	Set string	Set string	String: process_Step_2 = cleaning cassette (const.)	
2023-06-29 12:00:06	CXY-SM2	CXY-SM2	Valve switched to direction 2-3	
2023-06-29 12:00:07	CXY-SM1	CXY-SM1	Valve switched to direction 1-3	
2023-06-29 12:00:07	CXW-SM1	CXW-SM1	Valve switched to direction 1-3	
2023-06-29 12:00:07	CXW-SM3	CXW-SM3	Valve switched to direction 1-3	
2023-06-29 12:00:07	CXY-SM3	CXY-SM3	Valve switched to direction 1-2	
2023-06-29 12:00:07	CXX-SM1	CXX-SM1	Valve switched to direction 1-2	
2023-06-29 12:00:07	CXW-SM2	CXW-SM2	Valve switched to direction 2-3	
2023-06-29 12:00:13	EXH-SY	EXH-SY	Syringe starts, volume: 3000 ul, speed: 500 ul/s	
2023-06-29 12:00:16	timer_1	Timer 1	Wait 2.00 s	
2023-06-29 12:00:16	CXX-SM2	CXX-SM2	Valve switched to direction 1-2	
2023-06-29 12:00:16	CXX-SM1	CXX-SM1	Valve switched to direction 1-3	
2023-06-29 12:00:22	EXH-SY	EXH-SY	Syringe starts, volume: 3000 ul, speed: 500 ul/s	
2023-06-29 12:00:24	timer_1	Timer 1	Wait 2.00 s	
2023-06-29 12:00:24	CXW-SM2	CXW-SM2	Valve switched to direction 1-2	
2023-06-29 12:00:45	EXH-SY	EXH-SY	Syringe starts, volume: -6000 ul, speed: 300 ul/s	
2023-06-29 12:00:45	CXX-SM1	CXX-SM1	Valve switched to direction 1-2	
2023-06-29 12:00:45	CXW-SM2	CXW-SM2	Valve switched to direction 2-3	
2023-06-29 12:00:51	EXH-SY	EXH-SY	Syringe starts, volume: 3000 ul, speed: 500 ul/s	
2023-06-29 12:00:51	CXX-SM2	CXX-SM2	Valve switched to direction 1-2	
2023-06-29 12:00:51	CXX-SM1	CXX-SM1	Valve switched to direction 1-3	
2023-06-29 12:00:58	EXH-SY	EXH-SY	Syringe starts, volume: 3000 ul, speed: 500 ul/s	
2023-06-29 12:00:58	CXY-SM2	CXY-SM2	Valve switched to direction 1-2	
2023-06-29 12:00:58	CXY-SM3	CXY-SM3	Valve switched to direction 1-3	
2023-06-29 12:01:18	EXH-SY	EXH-SY	Syringe starts, volume: -6000 ul, speed: 300 ul/s	
2023-06-29 12:01:19	CXY-SM3	CXY-SM3	Valve switched to direction 1-2	
2023-06-29 12:01:19	CXY-SM2	CXY-SM2	Valve switched to direction 1-3	
2023-06-29 12:01:19	Set string	Set string	String: process_Step_2 = cleaning finished (const.)	
2023-06-29 12:01:19	Set string	Set string	String: process_Step_2 = NaCl to cool RV (const.)	
2023-06-29 12:01:19	CXX-SM1	CXX-SM1	Valve switched to direction 1-2	
2023-06-29 12:01:19	CXY-SM3	CXY-SM3	Valve switched to direction 1-2	
2023-06-29 12:01:19	CXW-SM3	CXW-SM3	Valve switched to direction 1-3	

Batch Report (cont.)

Timestamp	Object ID	Object Name	Process Step	Comment
2023-06-29 12:01:19	CXW-SM2	CXW-SM2	Valve switched to direction 2-3	
2023-06-29 12:01:29	EXH-SY	EXH-SY	Syringe starts, volume: 3000 ul, speed: 300 ul/s	
2023-06-29 12:01:34	timer_1	Timer 1	Wait 5.00 s	
2023-06-29 12:01:34	CXX-SM1	CXX-SM1	Valve switched to direction 1-3	
2023-06-29 12:01:34	CXX-SM2	CXX-SM2	Valve switched to direction 1-2	
2023-06-29 12:01:41	EXH-SY	EXH-SY	Syringe starts, volume: 3000 ul, speed: 500 ul/s	
2023-06-29 12:01:43	timer_1	Timer 1	Wait 2.00 s	
2023-06-29 12:01:43	CXX-SM2	CXX-SM2	Valve switched to direction 1-3	
2023-06-29 12:01:43	CXY-SM3	CXY-SM3	Valve switched to direction 1-3	
2023-06-29 12:01:43	Set string	Set string	String: process_Step_2 = (const.)	
2023-06-29 12:01:43	Set bit	Set bit	Bit: parallel tsk complete was set to: 1, (const.)	
2023-06-29 12:10:03	timer_1	Timer 1	Wait 500.00 s	
2023-06-29 12:10:06	timer_2	reaction time	Wait(var.) 600.00 s	
2023-06-29 12:10:07	EXC-HR	EXC-HR	normal mode, Wait 200 ms Tsp(var.): 80 C	
2023-06-29 12:10:07	Set string	Set string	String: process_step = cooling RV (const.)	
2023-06-29 12:10:07	Set string	Set string	String: process_Step_2 = (const.)	
2023-06-29 12:10:07	CXY-SM1	CXY-SM1	Valve switched to direction 1-2	
2023-06-29 12:10:07	CXY-SM2	CXY-SM2	Valve switched to direction 1-3	
2023-06-29 12:10:07	CXY-SM3	CXY-SM3	Valve switched to direction 1-3	
2023-06-29 12:10:07	CXW-SM3	CXW-SM3	Valve switched to direction 1-3	
2023-06-29 12:10:07	CXW-SM2	CXW-SM2	Valve switched to direction 2-3	
2023-06-29 12:10:07	EXC-HR	EXC-HR	normal mode, Wait 200 ms Tsp: 0 C	
2023-06-29 12:10:23	EXH-SY	EXH-SY	Syringe starts, volume: -6000 ul, speed: 400 ul/s	
2023-06-29 12:10:23	EXC-HR	EXC-HR	normal mode, Wait 200 ms Tsp(var.): 80 C	
2023-06-29 12:10:23	Set string	Set string	String: process_Step_2 = crude pro to HLB (const.)	
2023-06-29 12:10:23	EXC-HR	EXC-HR	normal mode, Wait 200 ms Tsp: 0 C	
2023-06-29 12:10:49	EXH-SY	EXH-SY	Syringe starts, volume: 9000 ul, speed: 350 ul/s	
2023-06-29 12:10:50	EXC-HR	EXC-HR	normal mode, Wait 200 ms Tsp: 0 C	
2023-06-29 12:10:50	CXY-SM3	CXY-SM3	Valve switched to direction 1-2	
2023-06-29 12:10:50	CXX-SM1	CXX-SM1	Valve switched to direction 1-3	
2023-06-29 12:10:50	CXX-SM2	CXX-SM2	Valve switched to direction 1-3	
2023-06-29 12:10:50	CXX-SM3	CXX-SM3	Valve switched to direction 1-3	
2023-06-29 12:10:50	CXZ-SM3	CXZ-SM3	Valve switched to direction 1-3	
2023-06-29 12:10:50	CXZ-SM2	CXZ-SM2	Valve switched to direction 1-3	
2023-06-29 12:10:50	CXZ-SM1	CXZ-SM1	Valve switched to direction 1-2	
2023-06-29 12:11:35	EXH-SY	EXH-SY	Syringe starts, volume: -9000 ul, speed: 200 ul/s	
2023-06-29 12:11:35	CXX-SM1	CXX-SM1	Valve switched to direction 1-2	
2023-06-29 12:11:50	EXH-SY	EXH-SY	Syringe starts, volume: 5000 ul, speed: 350 ul/s	
2023-06-29 12:11:55	timer_1	Timer 1	Wait 5.00 s	
2023-06-29 12:11:55	CXX-SM1	CXX-SM1	Valve switched to direction 1-3	
2023-06-29 12:11:55	CXX-SM2	CXX-SM2	Valve switched to direction 1-2	
2023-06-29 12:12:10	EXH-SY	EXH-SY	Syringe starts, volume: 5000 ul, speed: 350 ul/s	
2023-06-29 12:12:15	timer_1	Timer 1	Wait 5.00 s	
2023-06-29 12:12:15	CXX-SM2	CXX-SM2	Valve switched to direction 1-3	
2023-06-29 12:12:49	EXH-SY	EXH-SY	Syringe starts, volume: -10000 ul, speed: 300 ul/s	
2023-06-29 12:12:54	timer_1	Timer 1	Wait 5.00 s	
2023-06-29 12:13:04	timer_1	Timer 1	Wait 10.00 s	
2023-06-29 12:13:04	PCtrl	Flow Control	Msg: Ok to transfer product to vial?	
2023-06-29 12:13:04	Stop	Stop	Button switched from off to on	
2023-06-29 12:13:12	PCtrl	Flow Control	Msg was confirmed	
2023-06-29 12:13:12	Stop	Stop	Button switched from on to off	
2023-06-29 12:13:13	Set string	Set string	String: process_Step_2 = EtOH to HLB (const.)	
2023-06-29 12:13:13	CXX-SM3	CXX-SM3	Valve switched to direction 1-2	
2023-06-29 12:13:13	CXZ-SM3	CXZ-SM3	Valve switched to direction 1-2	
2023-06-29 12:13:23	EXH-SY	EXH-SY	Syringe starts, volume: 3000 ul, speed: 300 ul/s	
2023-06-29 12:13:25	timer_1	Timer 1	Wait 2.00 s	
2023-06-29 12:13:25	CXX-SM3	CXX-SM3	Valve switched to direction 1-3	
2023-06-29 12:13:38	EXH-SY	EXH-SY	Syringe starts, volume: -1200 ul, speed: 100 ul/s	
2023-06-29 12:13:58	timer_1	Timer 1	Wait 20.00 s	
2023-06-29 12:13:58	CXX-SM2	CXX-SM2	Valve switched to direction 1-2	
2023-06-29 12:14:04	EXH-SY	EXH-SY	Syringe starts, volume: 3000 ul, speed: 500 ul/s	
2023-06-29 12:14:04	CXY-SM3	CXY-SM3	Valve switched to direction 1-3	
2023-06-29 12:14:04	CXY-SM2	CXY-SM2	Valve switched to direction 1-2	
2023-06-29 12:14:04	CXZ-SM1	CXZ-SM1	Valve switched to direction 1-3	
2023-06-29 12:14:14	EXH-SY	EXH-SY	Syringe starts, volume: -4800 ul, speed: 500 ul/s	

Batch Report (cont.)

Timestamp	Object ID	Object Name	Process Step	Comment
2023-06-29 12:14:24	timer_1	Timer 1	Wait 10.00 s	
2023-06-29 12:14:24	CXY-SM3	CXY-SM3	Valve switched to direction 1-2	
2023-06-29 12:14:24	Set string	Set string	String: process_step = dilute product (const.)	
2023-06-29 12:14:24	CXX-SM1	CXX-SM1	Valve switched to direction 1-2	
2023-06-29 12:14:24	CXY-SM3	CXY-SM3	Valve switched to direction 1-2	
2023-06-29 12:14:42	EXH-SY	EXH-SY	Syringe starts, volume: 8500 ul, speed: 500 ul/s	
2023-06-29 12:14:47	timer_1	Timer 1	Wait 5.00 s	
2023-06-29 12:14:47	CXX-SM2	CXX-SM2	Valve switched to direction 1-3	
2023-06-29 12:14:47	CXX-SM1	CXX-SM1	Valve switched to direction 1-3	
2023-06-29 12:14:47	CXX-SM2	CXX-SM2	Valve switched to direction 1-3	
2023-06-29 12:15:30	EXH-SY	EXH-SY	Syringe starts, volume: -8500 ul, speed: 200 ul/s	
2023-06-29 12:15:40	timer_1	Timer 1	Wait 10.00 s	
2023-06-29 12:15:40	CXX-SM2	CXX-SM2	Valve switched to direction 1-2	
2023-06-29 12:15:54	EXH-SY	EXH-SY	Syringe starts, volume: 7000 ul, speed: 500 ul/s	
2023-06-29 12:15:54	CXX-SM2	CXX-SM2	Valve switched to direction 1-3	
2023-06-29 12:16:18	EXH-SY	EXH-SY	Syringe starts, volume: -7000 ul, speed: 300 ul/s	
2023-06-29 12:16:18	CXZ-SM3	CXZ-SM3	Valve switched to direction 1-3	
2023-06-29 12:16:18	CXZ-SM2	CXZ-SM2	Valve switched to direction 1-3	
2023-06-29 12:16:18	CXZ-SM1	CXZ-SM1	Valve switched to direction 1-2	
2023-06-29 12:16:18	Set string	Set string	String: process_step = process finished (const.)	
2023-06-29 12:16:18	Set string	Set string	String: process_Step_2 = remove product (const.)	
2023-06-29 12:16:18	PCtrl	Flow Control	Batch control: stop audit trail	
2023-06-29 12:16:19	PCtrl	Flow Control	Batch control: close batch	

Charts:

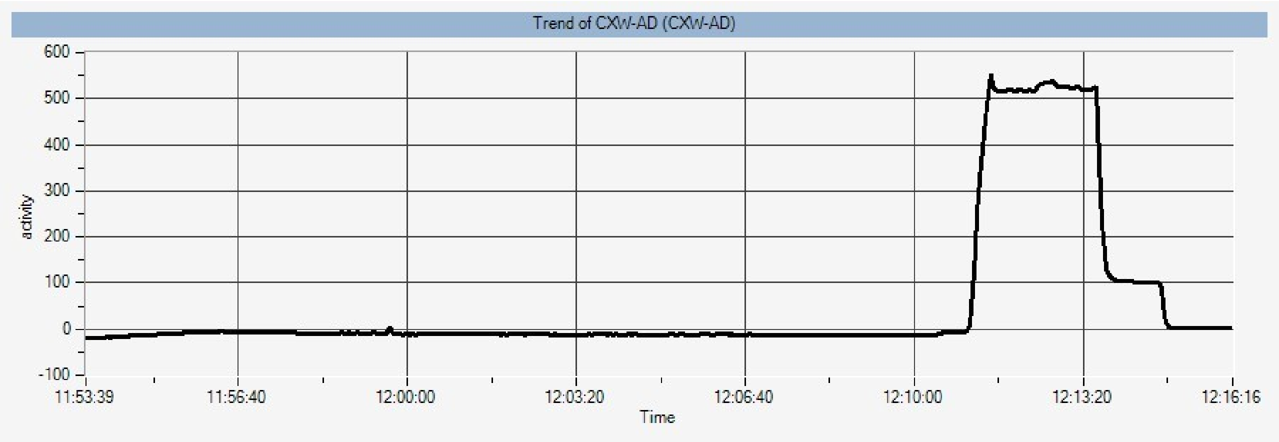

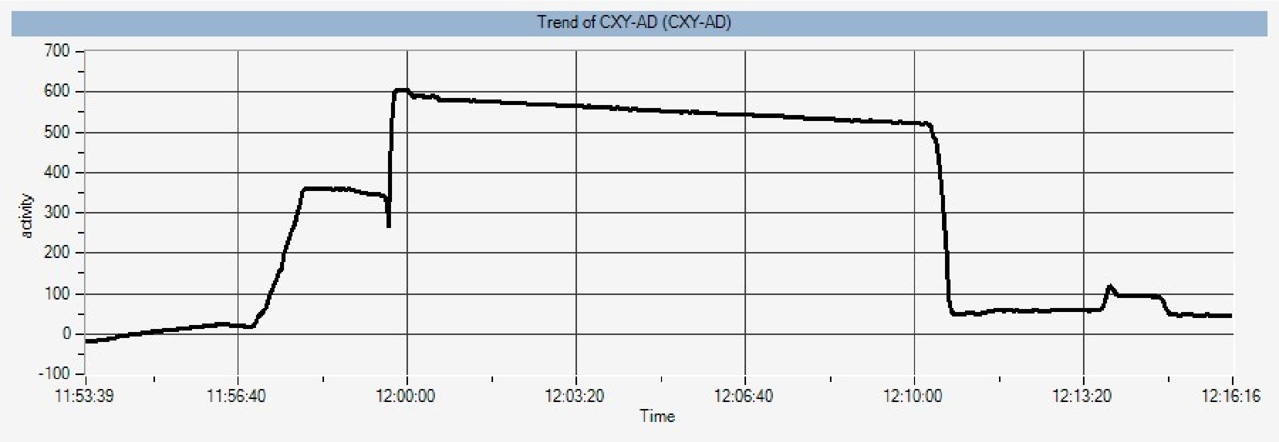

Batch Report (cont.)

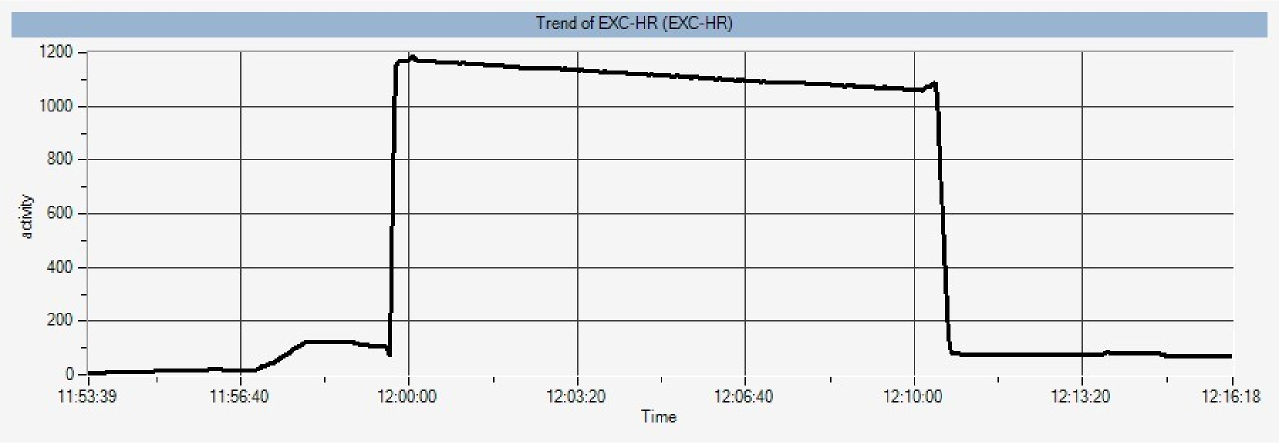

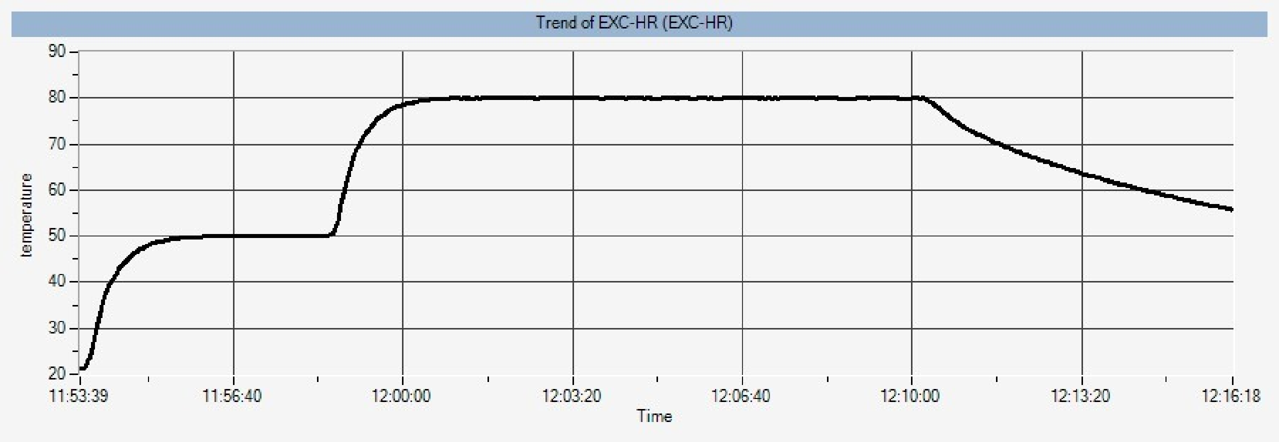

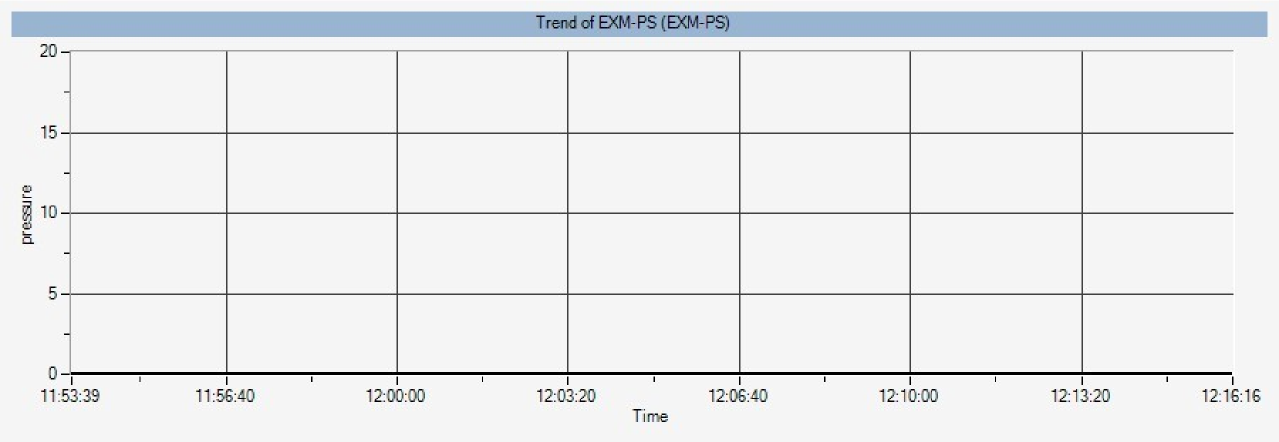

Supplement: Supplementary file 1 — Additional file 1. Exemplified batch report. [file 41181_2023_226_MOESM1_ESM.pdf]
