# Supplementary material for: Optimized, automated and cGMP-compliant synthesis of the HER2 targeting [68Ga]Ga-ABY-025 tracer
Source: EJNMMI Radiopharm Chem. 2023 Nov 22;8:41. doi: 10.1186/s41181-023-00226-y (PMC10665286; doi:10.1186/s41181-023-00226-y)
Supplement: Supplementary file 2 — Additional file 2. Table S1. Complete list of consumables and chemicals used. [file 41181_2023_226_MOESM2_ESM.docx]

# Supplementary material: Table S1

## Optimized, automated and cGMP-compliant synthesis of the HER2 targeting [^68^Ga]Ga-ABY-025 tracer

Emma Jussing^1,2*^, Mélodie Ferrat^1,2^, Mohammad M Moein^1,2^, Henrik Alfredéen^1,2^, Tetyana Tegnebratt^1^, Klas Bratteby^1,2^, Erik Samén^1,2^, Joachim Feldwish^4^, Renske Altena^2,3^, Rimma Axelsson^5,6^, Thuy A Tran^1,2^

1. Department of Radiopharmacy, Karolinska University Hospital, SE-171 76 Stockholm, Sweden
2. Department of Oncology and Pathology, Karolinska Institutet, SE-171 77 Stockholm, Sweden
3. Karolinska Comprehensive Cancer Center, Karolinska University Hospital, SE-171 77 Stockholm, Sweden
4. Affibody AB, SE-171 65, Solna, Sweden
5. Department of Medical Radiation Physics and Nuclear Medicine, Karolinska University Hospital, SE-171 76 Stockholm, Sweden
6. Department of Molecular Medicine and Surgery, Karolinska Institutet, SE-171 77 Stockholm, Sweden

***Corresponding author:** Emma Jussing, PhD, [emma.jussing@ki.se](mailto:emma.jussing@ki.se)

| **Table S1.** Complete list of consumables and chemicals used | | |
| --- | --- | --- |
| **Name** | **Article no.** | **Manufacturer** |
| ABY-025 precursor and reference standard, 0.5 mg, 1 mg/mL | 16188 | Affibody AB (supplier) |
| Modular-Lab cassette pack | C4-GA-PEP | Eckert & Ziegler |
| Modular-Lab reagent set  Vial 1: NaCl 5 M/HCl 0.13 M  Vial 2: Sodium acetate trihydrate 680 mg  Vial 2a: Acetic acid 0.35 mL, HCl 30% 0.15 mL, water 6.30 mL  Vial 2b: TraceSELECT water (not used for [^68^Ga]Ga-ABY-025)  Vial 3: EtOH/water 1:1  Vial 4: NaCl 9 mg/mL | EZ-102 | Eckert & Ziegler |
| GalliaPharm generator | 10056-00 | Eckert & Ziegler |
| Sterile Ultrapure 0.1 N Hydrochloric acid | 10027-00 | Eckert & Ziegler |
| Oasis hydrophilic-lipophilic balanced (HLB) plus light cartridge (with 30 mg sorbent) | 186005125 | Waters |
| FILL-EASE^TM^ Sterile vacuum vial, 15 mL | SVV-15C | Huayi |
| Millex-GV 0.22 µm/Ø33 sterile product filters | SLGV033RB | Merck, Millipore |
| Millex-FG 0.22 µm/Ø33 mm sterile vent filters | SLFG025LS | Merck, Millipore |
| Product transfer line, Tefzel® ETFE Tubing, 0.062” OD x 0.030” ID x 200 m, Spooled | S-15880 | GE Healthcare |
| Sterile water for injection | 128926 | B. Braun |
| Sterile ethanol (70%) | 1122 | Solveco |
| HiQ Nitrogen 6.0 | 136 | AGA |
| HiQ Helium 6.0 | 116 | AGA |
| HiQ Hydrogen 6.0 | 126 | AGA |
| Acetonitrile, gradient grade ≥ 99.9 % | 20060.320 | VWR |
| Trifluoroacetic acid, HPLC grade, ≥ 99% | 302031 | Sigma-aldrich |
| iTLC-SG chromatography paper | SGI0001 | Agilent |
| pH paper, pH 4.0-7.0 Mquant® | 1.09542.0001 | VWR |
| Citric acid monohydrate | 100244 | Merck |
| Sodium citrate dihydrate | W302600 | Sigma-Aldrich |
| 18 MΩ water for QC mobile phases | N.A. | In-house, via MilliQ |
